# Integrated transcriptomics and machine learning reveal diagnostic biomarkers and immune–stromal remodeling in ischemic heart failure

**DOI:** 10.3389/fbinf.2026.1822029

**Published:** 2026-03-31

**Authors:** Yang Sun, Yu Chang, Yezhi Feng, Binghui Song, Yanan Zhou

**Affiliations:** 1 Department of Cardiology, Qiqihar First Hospital, Qiqihar, Heilongjiang, China; 2 College of Basic Medical Sciences, Harbin Medical University, Harbin, Heilongjiang, China

**Keywords:** diagnostic biomarkers, immune microenvironment, ischemic heart failure, machine learning, transcriptomics

## Abstract

**Background:**

Ischemic heart failure (IHF) is a major cause of cardiovascular morbidity worldwide, characterized by complex tissue remodeling and inflammation. However, reliable molecular biomarkers for early diagnosis and a systematic understanding of the associated immune–stromal microenvironment remain limited. Identifying specific transcriptomic signatures may enhance diagnostic precision and reveal novel therapeutic targets.

**Methods:**

An integrative transcriptomic analysis was performed utilizing IHF datasets from the Gene Expression Omnibus (GEO). Differential expression analysis and Weighted Gene Co-expression Network Analysis (WGCNA) were employed to identify key disease-associated modules. To construct a robust diagnostic model, candidate features were screened using the intersection of four complementary machine learning algorithms: Least Absolute Shrinkage and Selection Operator (LASSO), Random Forest (RF), Support Vector Machine-Recursive Feature Elimination (SVM-RFE), and eXtreme Gradient Boosting (XGBoost). The immune and stromal landscape of IHF was comprehensively characterized using a hybrid approach combining MCP-counter and ssGSEA algorithms to quantify cell-type–specific infiltration patterns.

**Results:**

Through the integration of machine learning strategies, a robust 6-gene diagnostic signature was identified, comprising FCN3, OGN, ITPK1, HMOX2, MTCH1, and HMGN2. Immune deconvolution analysis revealed pronounced remodeling of the IHF microenvironment, characterized by significantly elevated infiltration of Endothelial cells, Macrophages, Neutrophils, and Natural killer cells, indicating a pro-inflammatory and angiogenic phenotype.

**Conclusion:**

This study identifies a novel and robust 6-gene diagnostic signature for Ischemic heart failure through a multi-algorithm machine learning framework. These biomarkers are intrinsically linked to pathological alterations in the cardiac stromal and immune microenvironment, particularly fibrosis and innate immune activation. Our findings provide a systems-level view of IHF pathogenesis and offer potential molecular targets for improved diagnosis and therapeutic intervention.

## 1 Introduction

Heart failure (HF) affects more than 37.7 million individuals worldwide (Ziaeian and Fonarow, 2016; Roger, 2021). With the ongoing global demographic shift toward an aging population, the anticipated surge in the burden of heart failure is poised to be considerable in the forthcoming years (Murphy et al., 2020). Ischemic heart failure (IHF) is a major subtype of HF characterized by chronic myocardial ischemia and irreversible myocardial damage (Hatamnejad et al., 2025; Manzi et al., 2025). The development and progression of IHF are highly complex and involve multiple pathophysiological processes, including persistent myocardial ischemia, cardiomyocyte loss, myocardial fibrosis, adverse ventricular remodeling, and sustained neurohormonal activation (Pastena et al., 2024; Marcos-Garcés et al., 2025). These mechanisms interact dynamically over time, ultimately leading to irreversible deterioration of cardiac structure and function. Despite advances in pharmacological therapy, device-based interventions, and comprehensive heart failure management, the prognosis of patients with IHF remains poor, with a 5-year mortality rate of 40%–50% (Benjamin et al., 2017; Bahit et al., 2018). Thus, IHF has become a substantial global health burden and an important research focus in cardiovascular medicine. Considering that the conventional biomarkers provide limited insight into the underlying molecular mechanisms of IHF and offer little guidance for the identification of novel therapeutic targets (Bastos et al., 2025). Therefore, the discovery of mechanism-driven biomarkers with higher specificity and biological interpretability remains an unmet clinical need.

With the rapid development of high-throughput transcriptomic technologies and bioinformatics approaches, large-scale gene expression profiling has become a powerful strategy for uncovering disease-associated molecular signatures. Previous bioinformatics studies of IHF have mainly relied on differential gene expression analysis combined with protein–protein interaction (PPI) network construction to identify candidate biomarkers (Jin et al., 2025). In recent years, weighted gene co-expression network analysis (WGCNA) has emerged as an effective systems biology method for identifying gene modules that are closely associated with clinical phenotypes. By constructing scale-free co-expression networks, WGCNA enables the identification of biologically meaningful gene clusters rather than isolated differentially expressed genes (Li et al., 2024). Furthermore, machine learning (ML) algorithms have been increasingly applied to biomedical research for feature selection and predictive model construction, offering improved robustness and classification performance (Jin et al., 2025).

**FIGURE 1:**
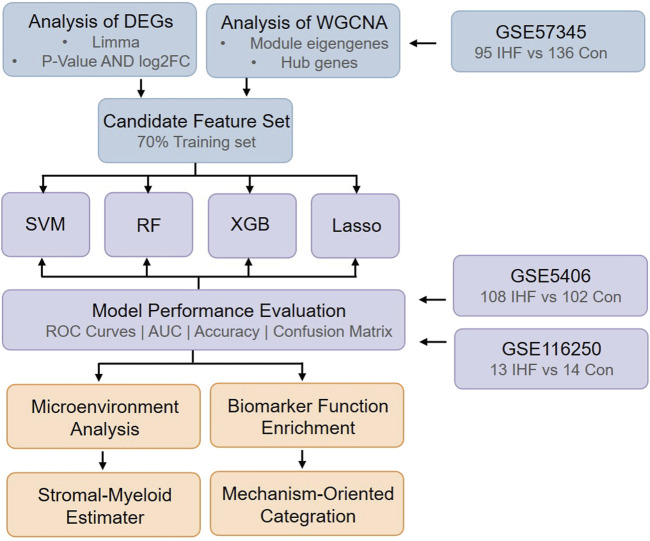
The study flowchart.

In this study, we first performed differential expression analysis using the GSE57345 dataset to characterize transcriptomic changes associated with ischemic heart failure (IHF). Then, WGCNA was applied to identify disease-related gene modules, and modules showing significant correlations with IHF status were selected for downstream analysis. Differentially expressed genes and hub genes derived from IHF-associated modules were subsequently integrated as candidate features. Based on these features, four supervised machine learning models—Least Absolute Shrinkage and Selection Operator (LASSO), Random Forest (RF), Support Vector Machine-Recursive Feature Elimination (SVM-RFE), and eXtreme Gradient Boosting (XGBoost)—were constructed and evaluated on an independent test set. All models showed stable classification performance, with LASSO achieving the highest overall discrimination ability. The RF model was further examined in detail to assess classification stability and feature contributions, and feature importance analysis revealed a multi-gene signature rather than reliance on a single dominant marker. To evaluate robustness, the reproducibility of RF-derived biomarkers was examined in the independent GSE5406 cohort, where consistent feature importance rankings were observed. In addition, expression patterns of top-ranked genes were assessed in the GSE116250 dataset, supporting cross-dataset stability at the expression level. Furthermore, immune and stromal cell infiltration analysis based on MCP-counter revealed coordinated alterations in myeloid and stromal compartments in IHF samples. Finally, functional annotation of RF-selected biomarkers showed strong enrichment in extracellular matrix organization, transcriptional regulation, cardiomyocyte structure, and stress response pathways, which was highly consistent with the observed immune–stromal remodeling. The overall analytical workflow and key findings are summarized in Figure 1. The code for this study is available at https://github.com/LuckyQ0Q/IHF_bioinformatics_analysis.git.

## 2 Materials and methods

### 2.1 Data collection and preprocessing

Three publicly available transcriptomic datasets related to ischemic heart failure (IHF) were manually downloaded from the Gene Expression Omnibus (GEO) database (Barrett et al., 2013). We chose GSE57345 (Liu et al., 2015; Jia et al., 2015) as the training dataset for ischemic heart failure (left ventricular myocardium samples from 95 patients with ischemic heart failure and 136 controls), also we chose GSE5406 (Hannenhalli et al., 2006) and GSE116250 (Sweet et al., 2018; Yamaguchi et al., 2020) for independent validation and external descriptive analysis. All datasets were preprocessed by R program (version 4.5.2) and ultimately harmonized at the Entrez Gene ID level to ensure cross-platform comparability. Then, we removed probes that did not correspond to gene symbols. Lastly, we eliminate the data’s batch effects. The basic information of all datasets was shown in Table 1.

**TABLE 1:** The details of datasets.

Series	Platforms	Samples	Tissue
GSE5406	GPL96	108 IHF and 102 Non-IHF	Left ventricle
GSE57345	GPL11532	95 IHF and 136 Non-IHF	Left ventricle
GSE116250	GPL16791	13 IHF and 14 Non-IHF	Left ventricle

### 2.2 Differential expression analysis and expression preprocessing

Expression data from GSE5406, GSE57345, and GSE116250 were classified according to curated phenotype annotations, retaining IHF and Non-IHF groups, while other disease subtypes were excluded. Differential expression analysis was performed using limma (Ritchie et al., 2015), and set | log2 (fold change) |≥0.5 and adjusted *p* < 0.05 as the threshold for differentially expressed genes (DEGs).

### 2.3 Weighted gene co-expression network analysis

Weighted gene co-expression network analysis (WGCNA) was conducted on the normalized GSE57345 expression matrix to identify gene modules associated with IHF. An unsigned adjacency matrix was constructed using a soft-thresholding power selected based on scale-free topology criteria, and gene modules were identified using the blockwiseModules function (minimum module size = 30, module merging cut height = 0.25). Module eigengenes were correlated with IHF status to detect biologically relevant modules (|correlation| >0.1, P < 0.05). Within significant modules, hub genes were defined by |gene significance| >0.2 and |module membership| >0.7, providing candidate genes for downstream integrative analyses.

### 2.4 Functional annotation of machine learning–derived biomarkers

Functional annotation was performed using Gene Ontology (GO) and Kyoto Encyclopedia of Genes and Genomes (KEGG) pathway analyses to classify genes into biological processes, molecular functions, and cellular components, focusing on categories like extracellular matrix organization, stress and injury response, cardiomyocyte integrity, and transcriptional or signaling regulation (Wu et al., 2021; Yu and He, 2016; Xu et al., 2024). This annotation provided a structured understanding of the biomarkers in the context of ischemic heart failure pathophysiology.

### 2.5 Machine learning–based biomarker identification and model evaluation

Machine learning–based analyses were performed using the GSE57345 dataset to identify transcriptomic biomarkers associated with IHF. Candidate features were defined as the union of differentially expressed genes from IHF-associated WGCNA modules. These genes were extracted to construct a sample-by-feature expression matrix. Samples were randomly divided into training and testing sets at a 7:3 ratio using stratified sampling. Four supervised machine learning models (LASSO, RF, SVM-RFE and XGBoost) were trained on the training set and evaluated on the independent test set. RF feature importance was estimated using the mean decrease in Gini index, LASSO feature selection was performed via cross-validated regularization, XGBoost was implemented using a binary logistic objective, and SVM was constructed using a radial basis function (RBF) kernel to capture potential non-linear relationships between gene expression features and disease status (Teja and Rayalu, 2025; Guo et al., 2021). Model performance was assessed using area under the receiver operating characteristic curve (AUC) and classification accuracy, with ROC curves generated for comparative evaluation. The final candidate biomarkers were selected by intersecting the feature genes identified by the four machine learning algorithms.

### 2.6 Independent validation and external descriptive analysis

To assess the robustness and reproducibility of machine learning–derived biomarkers, independent validation was performed using the GSE5406 dataset to examine the reproducibility of candidate biomarkers identified in the discovery cohort. Candidate biomarkers identified from the discovery cohort through WGCNA and machine learning–based feature selection were extracted from the validation datasets. To further evaluate the generalizability of these biomarkers across independent cohorts, an additional external dataset (GSE116250) was included for validation. Gene identifiers were converted to gene symbols where necessary to ensure consistency across datasets. In both validation datasets, expression distributions of candidate biomarkers between IHF and non-failing control samples were visualized using violin plots, and statistical significance was assessed using the Wilcoxon rank-sum test. Receiver operating characteristic (ROC) curve analysis was performed to evaluate the diagnostic performance of each biomarker.

### 2.7 Stromal–myeloid microenvironment analysis

The stromal–myeloid microenvironment in IHF was characterized using the hybrid computational strategy combining MCP-counter (Becht et al., 2016) and single-sample gene set enrichment analysis (ssGSEA) (Barbie et al., 2009) based on transcriptomic data from the GSE57345 dataset. Gene identifiers were converted from Entrez IDs to gene symbols using the *org. Hs.eg.db* package, and genes without valid annotations were excluded. MCP-counter was applied to estimate the relative abundance of major non-lymphoid cell populations that are robustly captured in bulk cardiac tissue, including fibroblasts, endothelial cells, monocytic lineage cells, and neutrophils. To complement this analysis and improve resolution for immune cell subsets that are underrepresented in MCP-counter, ssGSEA was performed using curated immune cell marker gene sets to infer enrichment scores for additional immune populations, such as macrophages, NK cells, mast cells, and T-cell subsets.

To facilitate cross-sample comparison, cell-type scores were Z-score normalized within each population and group-level differences were quantified by calculating the difference in mean Z-scores (ΔZ-score) between groups for each cell type.

## 3 Results

### 3.1 Identification of differentially expressed genes and IHF-Related modules

**FIGURE 2:**
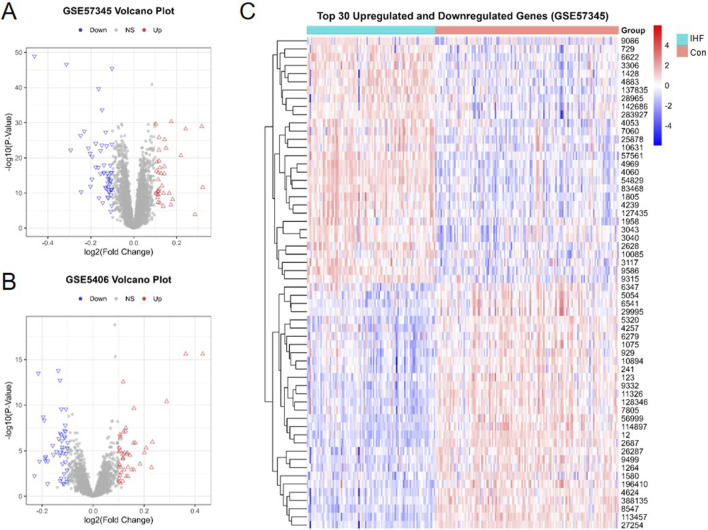
Identification of (DEGs in IHF. **(A,B)** Volcano plot identifying DEGs in the dataset GSE57345 and GSE5406. Red dots represent upregulated genes, and blue dots represent downregulated genes. **(C)** A heatmap showing the top 30 upregulated and the top 30 downregulated genes in IHF and non-failing control samples.

To elucidate the transcriptomic alterations associated with IHF, differential expression analysis was initially performed on the datasets. A total of **88** DEGs were identified between IHF and non-failing control samples in the GSE57345 dataset, including **35** upregulated and **52** downregulated genes (Figure 2A). A similar volcano plot visualization was performed for the validation dataset GSE5406 to observe the distribution of DEGs (Figure 2B). Then, we analyzed the expression patterns of the most significant alterations and generated a hierarchical clustering heatmap of the top 30 upregulated and downregulated DEGs. Thus, the distinct transcriptional profiles between IHF and non-failing control samples were revealed, confirming the robustness of the identified DEGs (Figure 2C).

**FIGURE 3:**
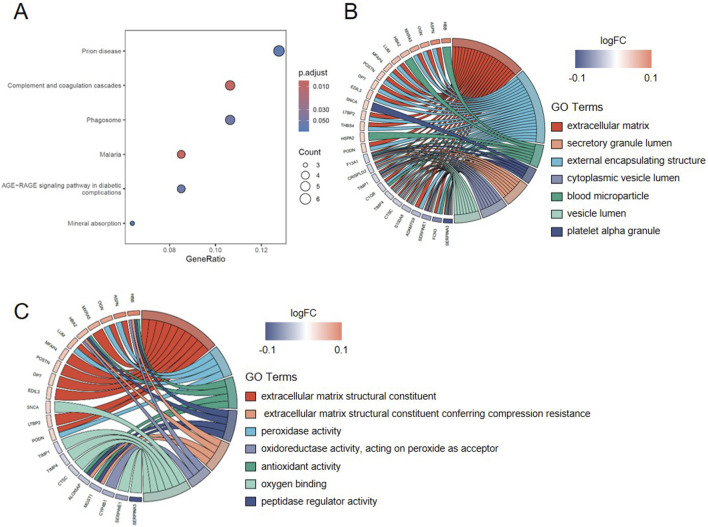
Functional enrichment analysis of DEGs. **(A)** Bubble plot of significant KEGG pathways. The size of the bubble represents the gene count, and the color gradient (blue to red) indicates statistical significance (P-adjust). **(B,C)** Chord plots visualizing the relationship between DEGs and enriched GO terms. Genes (left semicircle) are linked to their associated functional terms (right semicircle).

Subsequently, to explore the biological functions and signaling pathways underlying these DEGs, we also performed GO and KEGG pathway analysis to investigate the biological function of DEGs. KEGG pathway analysis indicated that the DEGs were significantly enriched in immune-inflammatory and signaling pathways, predominantly the Complement and coagulation cascades, Phagosome, and the AGE-RAGE signaling pathway in diabetic complications (Figure 3A). Furthermore, Gene Ontology (GO) analysis visualized by chord plots revealed the complex associations between DEGs and their functional terms. The DEGs were primarily localized to the extracellular matrix (ECM), secretory granule lumens, and cytoplasmic vesicle lumens (Figures 3B,C). These results suggest that IHF pathogenesis involves extensive extracellular matrix remodeling and activation of vesicle-mediated immune responses.

### 3.2 Construction of Co-Expression network and identification of key modules

**FIGURE 4:**
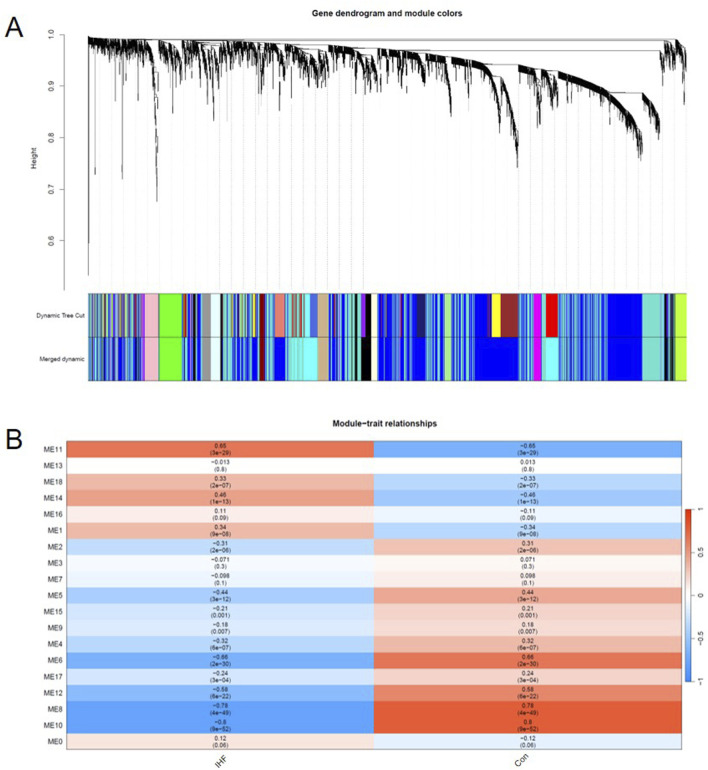
Weighted gene co-expression network analysis (WGCNA). **(A)** Dendrograms for gene and trait clustering in IHF were created. These gene clustering trees, or dendrograms, were derived from hierarchical clustering based on neighbor-related differences. **(B)** Module-trait relationship heatmap. Each cell within these modules displays the correlation coefficient and the corresponding p-value.

Weighted Gene Co-expression Network Analysis (WGCNA) was performed on the GSE57345 dataset to construct a gene co-expression network. To ensure a scale-free network topology, the optimal soft-thresholding power was set to *β* = 4. Based on this parameter, a hierarchical clustering tree was constructed to identify gene modules. As shown in Figure 4A, a total of 19 distinct co-expression modules were identified via the dynamic tree cutting algorithm.

Subsequently, module-trait relationship analysis was conducted to correlate these modules with the IHF phenotype. As illustrated in Figure 4B, several modules exhibited significant correlations with IHF status. Notably, the ME8 module showed the strongest positive correlation with IHF (correlation coefficient = 0.76, *P* = 4e-49), indicating that genes within this module are significantly upregulated in the disease group. In contrast, the ME11 module displayed the most significant negative correlation with IHF (correlation coefficient = −0.65, *P* = 3e-39). Consequently, the significant modules containing a total of 2,214 genes were selected as the key modules for further investigation.

### 3.3 Screening and verification of optimal diagnostic biomarkers via machine learning

**FIGURE 5:**
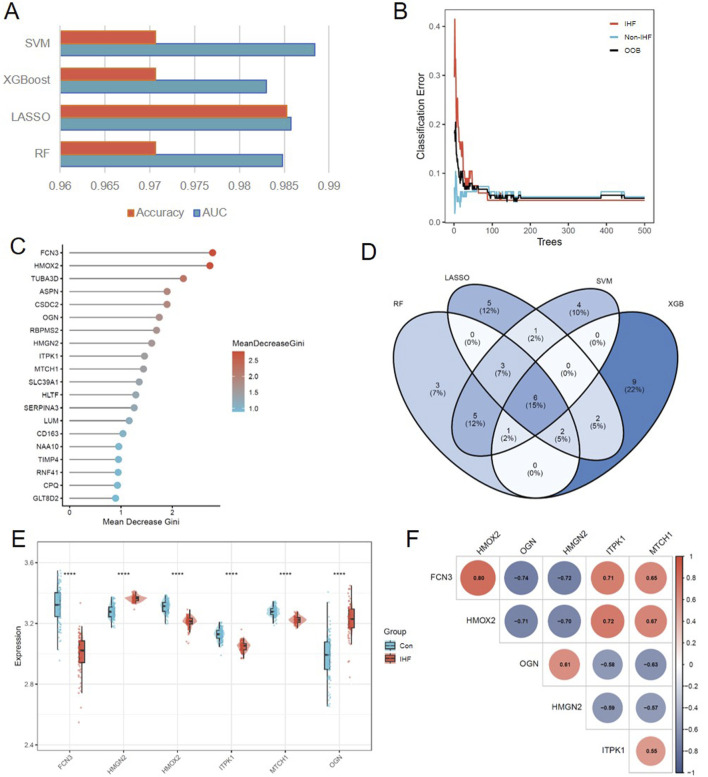
The result analysis of machine learning. **(A)** Performance comparison of four machine learning models based on Accuracy and AUC metrics. **(B)** The relationship between the number of trees and the classification error rate in the RF model. **(C)** Lollipop plot ranking the top 20 features based on Mean Decrease Gini importance in the RF model. **(D)** Venn diagram showing the intersection of characteristic genes identified by the four machine learning algorithms, resulting in 6 common biomarkers. **(E)** Violin plots visualizing the expression levels of the 6 optimal biomarkers in the IHF versus Control groups in the training set. Statistical significance was determined using the Wilcoxon test (****P < 0.000). **(F)** Correlation heatmap displaying the Spearman correlation coefficients among the 6 identified biomarkers. Red indicates positive correlation, and blue indicates negative correlation.

To identify robust and accurate diagnostic biomarkers for IHF, we integrated the DEGs and WGCNA-identified hub genes in previous steps into four distinct machine learning algorithms, including RF, LASSO, SVM and XGBoost. The predictive performance of these models was evaluated using the AUC and accuracy metrics. As illustrated in Figure 5A, all four models demonstrated excellent classification capabilities, with AUC values exceeding 0.98, indicating the high reliability of the candidate feature set.

Specifically, for the Random Forest model, the error rate stabilized as the number of trees increased, ensuring model robustness (Figure 5B). We subsequently ranked the features based on the Mean Decrease Gini index to visualize the relative importance of top candidate genes (Figure 5C). To further narrow down the targets and obtain the most consensus-based biomarkers, we intersected the optimal features selected by all four algorithms (RF, LASSO, SVM-RFE, and XGBoost). As shown in the Venn diagram (Figure 5D), a total of **6** overlapping genes were identified as the optimal diagnostic biomarkers: FCN3, HMOX2, OGN, HMGN2, ITPK1, and MTCH1.

Validation of these six biomarkers in the training dataset revealed statistically significant differences in expression levels between the IHF and control groups (P < 0.0001), confirming their dysregulation in the disease state (Figure 5E). Furthermore, a correlation analysis was performed to explore the interplay among these biomarkers. The results demonstrated strong co-expression patterns (Figure 5F); for instance, FCN3 exhibited a significant positive correlation with HMOX2 (r = 0.80) and ITPK1 (r = 0.71), but a negative correlation with HMGN2 (*r* = −0.72), suggesting potential functional synergy or antagonistic regulation in the pathogenesis of IHF.

### 3.4 Independent validation of optimal biomarkers in external datasets

To verify the robustness and reproducibility of the identified biomarkers, we performed independent validation using two external datasets: GSE5406 and GSE116250.

**FIGURE 6:**
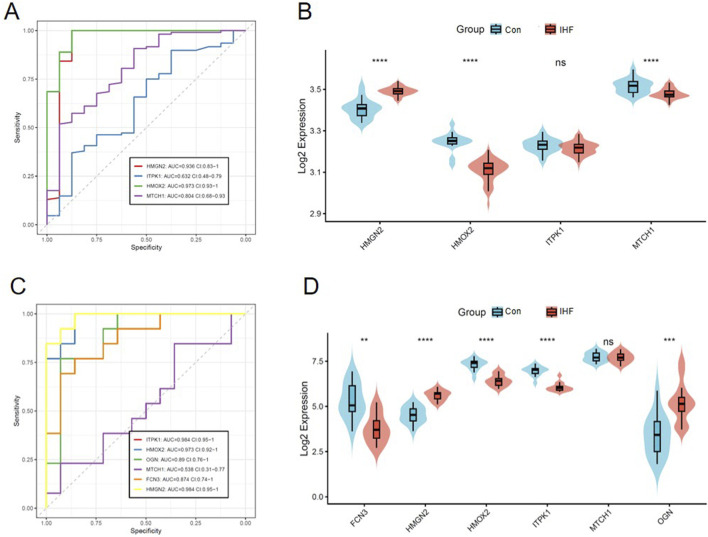
Independent validation of the optimal biomarkers in external datasets. **(A,B)** Validation in the GSE5406 dataset. ROC curves for 4 available biomarkers and Violin plots displaying the significant differential expression of these biomarkers in IHF vs. Control samples. **(C,D)** Validation in the GSE116250 dataset. ROC curves for all six optimal biomarkers and Violin plots displaying the significant differential expression of these biomarkers in IHF vs. Control samples. Statistical significance was determined using the Wilcoxon test (**P < 0.01, ***P < 0.001, ****P < 0.0001, ns: not significant).

First, in the GSE5406 dataset, four of the six optimal biomarkers (HMGN2, ITPK1, HMOX2, and MTCH1) were available for analysis due to platform-specific probe coverage. Receiver operating characteristic (ROC) curve analysis demonstrated that these genes maintained high diagnostic accuracy, with HMOX2 (AUC = 0.973) and HMGN2 (AUC = 0.936) exhibiting exceptional predictive performance (Figure 6A). Consistent with the training set, differential expression analysis confirmed that HMGN2 and MTCH1 were significantly upregulated, while HMOX2 was significantly downregulated in IHF samples compared to controls (*P* < 0.0001, Figure 6B). Although ITPK1 did not show statistical significance in expression difference in this specific dataset, its AUC value of 0.632 still suggested potential diagnostic utility.

Subsequently, we extended the validation to the larger GSE116250 dataset, where all six biomarkers were successfully detected. The ROC analysis revealed outstanding diagnostic capabilities for most genes, particularly ITPK1 (AUC = 0.984), HMGN2 (AUC = 0.984), and HMOX2 (AUC = 0.973), all achieving AUC values near unity (Figure 6C). Expression validation further corroborated the reliability of these biomarkers; FCN3, HMOX2, and ITPK1 were significantly downregulated, whereas HMGN2 and OGN were significantly upregulated in the IHF group (*P* < 0.01, Figure 6D). While MTCH1 showed no significant difference in this dataset, the overall consistency of the remaining biomarkers across multiple cohorts strongly supports their potential as reliable diagnostic signatures for IHF.

### 3.5 Integrated evaluation of the stromal–immune microenvironment

Given that ischemic injury triggers significant remodeling of the cardiac cellular landscape, we utilized a combinatorial approach leveraging MCP-counter and ssGSEA algorithms to quantify the abundance of six key stromal and immune cell populations. This analysis focused on endothelial cells and fibroblasts (stromal components), as well as macrophages, neutrophils, monocytic lineage cells, and natural killer (NK) cells (immune components), providing a comprehensive view of the tissue repair and inflammatory milieu.

**FIGURE 7:**
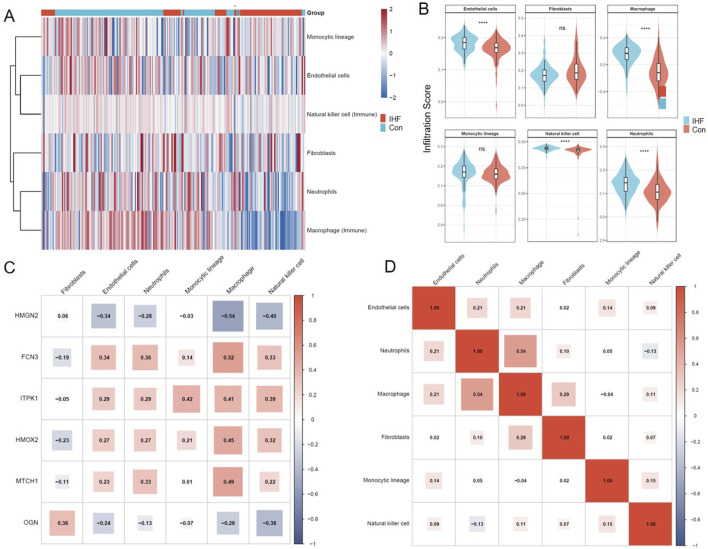
Evaluation of the immune and stromal microenvironment in IHF. **(A)** Heatmap visualizing the landscape of cellular infiltration for six key cell type. The color scale represents Z-score normalized infiltration scores. **(B)** Violin plots comparing the infiltration scores of six cell types between the IHF (blue) and Control (red) groups. Statistical significance was determined using the Wilcoxon rank-sum test (****P < 0.0001; ns: not significant). **(C)** Correlation heatmap showing the association between the expression levels of the six diagnostic biomarkers and the immune/stromal cells (columns). **(D)** Cellular co-expression matrix illustrating the crosstalk among the six infiltrating cell types.

The landscape of cellular composition revealed distinct microenvironmental heterogeneity between IHF and control samples (Figure 7A). Quantitative analysis further highlighted significant alterations in specific cellular infiltrates (Figure 7B). The IHF group exhibited a profoundly pro-inflammatory and angiogenic phenotype compared to controls. Specifically, the infiltration scores of Endothelial cells, Macrophages, Neutrophils, and Natural killer cells were all significantly elevated in IHF tissues (*P* < 0.0001). This elevation suggests a coordinated response involving compensatory angiogenesis alongside robust innate immune activation and cytotoxicity. Conversely, no statistically significant differences were observed in the overall abundance of Fibroblasts and Monocytic lineage cells between the two groups, implying that their functional states or specific subsets, rather than total abundance, might be more relevant in this cohort.

To explore the potential regulatory roles of the identified biomarkers in shaping this microenvironment, we performed a Spearman correlation analysis between the expression of the six optimal biomarkers and cellular infiltration scores (Figure 7C). The results uncovered distinct biomarker-specific microenvironmental associations. OGN (Osteoglycin) exhibited a unique positive correlation with Fibroblasts (*r* = 0.36) while showing negative associations with inflammatory cells, consistent with its established role in extracellular matrix organization and fibrosis. In contrast, FCN3, ITPK1, HMOX2, and MTCH1 displayed broad positive correlations with the inflammatory cluster, particularly with Macrophages and Neutrophils. Notably, FCN3 showed the strongest correlation with Macrophages (*r* = 0.52), suggesting its involvement in immune cell recruitment. Conversely, HMGN2 exhibited a distinct inverse correlation pattern with Macrophages (*r* = −0.54) and NK cells (*r* = −0.40), indicating a potential protective or immunomodulatory role.

Furthermore, we constructed a cellular co-expression network to visualize the interplay among these cell types (Figure 7D). A moderate positive correlation was observed between Macrophages and Neutrophils (*r* = 0.54), reflecting their synergistic recruitment during the acute and chronic phases of ischemic inflammation. Additionally, Macrophages showed a mild positive association with Fibroblasts (*r* = 0.29), hinting at the crosstalk between inflammation and fibrotic remodeling. Collectively, these findings indicate that the identified diagnostic biomarkers are intrinsically linked to the pathological alterations of the cardiac stromal and immune microenvironment.

## 4 Discussion

Ischemic heart failure (IHF) remains a leading cause of global mortality, arising from a complex interplay among cardiomyocyte death, maladaptive extracellular matrix (ECM) remodeling, oxidative stress, and immune dysregulation. Currently, circulating natriuretic peptides such as B-type natriuretic peptide (BNP) and N-terminal pro-BNP (NT-proBNP) are widely used for the diagnosis and prognosis of heart failure. However, these markers primarily reflect hemodynamic stress and cardiac wall stretch, and they provide limited insight into the underlying molecular remodeling processes within the myocardium. In this study, we applied a rigorous multi-step machine learning framework integrating transcriptomic profiling, weighted gene co-expression network analysis (WGCNA), and immune infiltration estimation to derive a robust 6-gene diagnostic signature (FCN3, OGN, ITPK1, HMOX2, MTCH1, and HMGN2). The gene panel identified in this study captures multiple pathophysiological dimensions of ischemic heart failure, including extracellular matrix remodeling, oxidative stress, mitochondrial dysfunction, and immune–stromal interactions. As a result, such transcriptomic biomarkers may offer complementary information for molecular stratification of patients and could potentially support the development of precision diagnostic strategies in the future.

A hallmark of IHF is the replacement of functional myocardium with fibrotic scar tissue, driven by dysregulated ECM synthesis and turnover. Our pathway enrichment analysis of the top dysregulated genes and the final diagnostic model highlighted profound alterations in ECM organization. Recent multi-omics studies of both dilated and ischemic cardiomyopathy have confirmed that ECM remodelling pathways are among the most significantly enriched in failing hearts, with elevated expression of collagen genes and matrix regulators in diseased myocardium (Portokallidou et al., 2023).

OGN (Osteoglycin), a member of the small leucine-rich proteoglycan family, emerged as a key biomarker in our model. Beyond its structural role, OGN has been implicated in regulating collagen fibrillogenesis and myocardial scar maturation, with recent biomarker studies confirming its elevation in advanced heart failure and linking it to adverse remodeling dynamics in human cohorts (Van Aelst et al., 2015; Yu et al., 2024). In line with our immune infiltration analysis, OGN correlated positively with fibroblast abundance (r = 0.36), consistent with its role as a stromal regulator rather than a classical immune effector.

FCN3 (Ficolin-3) also featured prominently in our signature and machine learning selection. Although historically studied as a circulating complement lectin pathway protein, recent transcriptomic analyses have associated FCN3 expression with heart failure phenotypes and inflammatory activation in ischemic cardiomyopathy datasets (Jiang et al., 2022; Chen et al., 2021). Complement pathway components, including lectin pathway initiators, have been implicated in cardiac inflammation and remodeling, supporting a “fibro-inflammatory” axis whereby FCN3 may modulate macrophage recruitment and innate immune activation in the failing myocardium (Hok et al., 2025).

The enrichment of other ECM-associated genes such as LUM (Lumican) among the top dysregulated set further validates the fibrotic signature captured by our model. Lumican has been mechanistically linked to proper collagen fibril assembly and myocardial stiffness in ischemic hearts through recent transcriptomic profiling, emphasizing the centrality of ECM perturbation in heart failure progression (Portokallidou et al., 2023).

In addition to structural remodeling, ischemic injury provokes oxidative stress and mitochondrial dysfunction. HMOX2 (Heme Oxygenase-2) emerged as a predictive marker in our model; unlike its stress-inducible isoform HMOX1, HMOX2 is constitutively expressed and contributes to baseline antioxidant defense by metabolizing heme into biliverdin and carbon monoxide. Disruption of this constitutive antioxidant tone may confer vulnerability to reactive oxygen species in chronically ischemic myocardium, consistent with human transcriptomic IHF signatures (Sties et al., 2018; Zhao S. T. et al., 2024).

In parallel, MTCH1 (Mitochondrial Carrier 1), a regulator of mitochondrial apoptosis signaling, was selected by multiple machine learning algorithms. While direct mechanistic studies in human hearts are scarce, the consistent selection of MTCH1 underscores mitochondrial apoptotic susceptibility as a discriminative molecular feature of ischemic myocardium, integrating metabolic dysfunction with cell death pathways (Zhao et al., 2024a).

Although our final diagnostic model focused on stable markers, analysis of the broader gene set revealed upstream transcriptional drivers (Supplementary Table S1). Immediate-early transcription factors such as EGR1 and FOS were prominently upregulated in ischemic samples, reflecting rapid stress responses. These regulators have been repeatedly identified in transcriptomic studies of reperfusion and chronic ischemia, supporting their role in orchestrating inflammation and stress signaling in IHF (Khachigian, 2006; Khachigian, 2024).

A unique strength of this study is the integration of diagnostic signatures with stromal and immune landscape profiling. Our results depict an IHF microenvironment marked by increased endothelial activity and innate immune activation (macrophages and neutrophils), consistent with chronic inflammatory signatures observed in large transcriptomic meta-analyses of ischemic cardiomyopathy (Li et al., 2018). Immune-stromal correlation patterns, such as the positive association of ITPK1 with endothelial and myeloid populations, suggest that metabolic regulators may intersect with immune activation in ischemic tissue remodeling. While ITPK1’s classical function is in inositol phosphate metabolism, its broader correlations point towards potential involvement in endothelial activation and leukocyte metabolic reprogramming under ischemic conditions. Similarly, the inverse correlation of HMGN2 (a nucleosome-binding protein) with macrophages and NK cells implicates chromatin regulation in modulating inflammatory dynamics, aligning with studies that identify HMGN2 as a heart failure signature gene with roles in stress and transcriptional homeostasis (Wang et al., 2024).

Despite rigorous validation across computational frameworks, our study has limitations. First, as a retrospective bioinformatic analysis, these findings require external cohort validation and experimental confirmation (e.g., RT-qPCR, Western blot, immunostaining) to establish causality and functional relevance. Second, computational immune infiltration estimates provide relative cell abundance; future work should integrate spatial and single-cell resolution to quantify absolute immune cell populations in ischemic myocardium. Furthermore, recent advances in computational biology have further highlighted the importance of integrating multi-omics data and advanced machine learning frameworks for biomarker discovery. For example, emerging studies have demonstrated the potential of combining diverse biological layers, including microbiome and metabolomics data, to improve disease prediction and mechanistic interpretation (Zhao et al., 2024a; Zhao et al., 2022). In parallel, recent work in clinical data science has proposed increasingly sophisticated machine learning architectures capable of integrating heterogeneous biomedical datasets to enhance predictive performance and interpretability (Zhao et al., 2024b; Zhang et al., 2026). Although the present study focused on transcriptomic data, future research integrating transcriptomics with other omics layers and advanced machine learning models may further refine biomarker discovery and improve the predictive power of diagnostic models in ischemic heart failure.

In summary, we have constructed a robust 6-gene diagnostic model for IHF that not only distinguishes disease status but also reflects core pathophysiological hallmarks—fibrotic remodeling (OGN, FCN3), oxidative stress (HMOX2), and mitochondrial dysregulation (MTCH1). By elucidating associations between these biomarkers and the immune-stromal landscape, our study advances understanding of the fibro-inflammatory mechanisms underpinning ischemic heart failure and highlights candidate targets for precision diagnostics and therapeutic intervention. Another important aspect of this study is the use of a multi-algorithm consensus strategy for biomarker identification. By integrating the results from multiple algorithms, including LASSO, RF, SVM-RFE, and XGBoost, the intersection approach helps identify features that are consistently selected across models rather than those favored by a single method. This consensus-based strategy can reduce the influence of model-specific variability and improve the robustness and stability of the identified biomarkers. Consequently, genes retained through the multi-algorithm intersection are more likely to represent biologically relevant and reproducible disease-associated signals.

## Data Availability

The original contributions presented in the study are included in the article/Supplementary Material, further inquiries can be directed to the corresponding author.
