# Supplementary material for: Integrated transcriptomics and machine learning reveal diagnostic biomarkers and immune–stromal remodeling in ischemic heart failure
Source: Front Bioinform. 2026 Mar 31;6:1822029. doi: 10.3389/fbinf.2026.1822029 (PMC13076298; doi:10.3389/fbinf.2026.1822029)
Supplement: Supplementary file 1 [file Table1.docx]

Supplementary Material

# Supplementary Tables

| **Symbol** | **Gene_Name** | **Functional_Category** |
| --- | --- | --- |
| FCN3 | ficolin 3 | Fibroblast / ECM organization |
| HLTF | helicase like transcription factor | Transcriptional / signaling regulation |
| EPN1 | epsin 1 | Cardiomyocyte structural integrity |
| OGN | osteoglycin | Fibroblast / ECM organization |
| NAA10 | N-alpha-acetyltransferase 10, NatA catalytic subunit | Other / Metabolic |
| EGR1 | early growth response 1 | Stress response / Injury signaling |
| MYOT | myotilin | Cardiomyocyte structural integrity |
| FOS | Fos proto-oncogene, AP-1 transcription factor subunit | Stress response / Injury signaling |
| HMOX2 | heme oxygenase 2 | Stress response / Injury signaling |
| TINAGL1 | tubulointerstitial nephritis antigen like 1 | Fibroblast / ECM organization |
| MTCH1 | mitochondrial carrier 1 | Cardiomyocyte structural integrity |
| MLLT1 | MLLT1 super elongation complex subunit | Transcriptional / signaling regulation |
| TMEM71 | transmembrane protein 71 | Other / Metabolic |
| POR | cytochrome p450 oxidoreductase | Stress response / Injury signaling |
| LUM | lumican | Fibroblast / ECM organization |
| ZNF25 | zinc finger protein 25 | Other / Metabolic |
| KCMF1 | potassium channel modulatory factor 1 | Other / Metabolic |
| SCYL1 | SCY1 like pseudokinase 1 | Transcriptional / signaling regulation |
| STK40 | serine/threonine kinase 40 | Transcriptional / signaling regulation |
| CPQ | carboxypeptidase Q | Other / Metabolic |

**Supplementary Table 1.** Functional categorization of the top 20 genes ranked by Random Forest feature importance. Genes were grouped according to their primary biological roles, including extracellular matrix organization, transcriptional or signaling regulation, cardiomyocyte structural integrity, stress response, and metabolic functions.
